# From haze to horizon: epigenetic research and artificial intelligence in child and adolescent psychiatry

**DOI:** 10.1007/s00787-025-02686-w

**Published:** 2025-03-20

**Authors:** Yulia Golub, Antje Wulff, Torsten Plösch

**Affiliations:** 1 Department of Child and Adolescent Psychiatry, Psychosomatic and Psychotherapy, School of Medicine and Health Sciences, Carl von Ossietzky Universität Oldenburg, Oldenburg, Germany; 2 Big Data in Medicine, Department of Health Services Research, Carl von Ossietzky Universität Oldenburg, Oldenburg, Germany; 3 Department of Human Medicine, Division of Perinatal Neurobiology, School of Medicine and Health Science, Carl von Ossietzky Universität Oldenburg, Oldenburg, Germany; 4 Department of Obstetrics and Gynaecology, University Medical Center Groningen, University of Groningen, Groningen, Netherlands

As decades before, methods for diagnosing child and adolescent psychiatric (CAP) disorders still largely depend on the collection of patient histories by physicians and self-reported questionnaires from patients, with CAP assessments being largely “expert-based diagnostic” and “expert-based treatment” decisions.

This is happening despite significant advances in our understanding of CAP risk and protective factors, environment-genome interactions, and the development of machine learning and artificial intelligence (AI) to help in for decision making.

How can we leverage this knowledge to enhance our understanding of CAP disorders, thereby advancing both clinical practice and scientific progress?

Numerous environmental risk and protective factors for CAP disorders, such as family interactions, peer relationships, school environment, significant life events, parental history, socioeconomic status, early life adversities, and many others have been described [9]. However, the mechanisms of how exactly, different CAP conditions can emerge from common, non-specific environmental influences remains ununderstood. The prevailing belief is that these environmental factors interact with other critical elements, such as a child’s genetic predispositions and developmental stage (i.e., the timing of exposure in relation to the child’s level of maturation) [1] to cause a CAP disorders.

Potential mechanisms through which the genome can ‘capture’ the effects of environmental exposures and propagate their influence are epigenetic processes, which are heritable changes in gene expression that do not involve alterations to the underlying DNA sequence. Of these, DNA methylation is currently the most widely investigated and best understood epigenetic process, involving the addition of methyl groups to DNA, which typically suppresses gene expression without altering the underlying genetic sequence (as reviewed by Greenberg & Bourc’his [5],.

Research has demonstrated (1) that DNA methylation is responsive to environmental factors starting in utero, such as dietary, chemical, and psychosocial exposures [8]; (2) is temporally dynamic, playing a critical role in (neuro)development [10]; (3) and that abnormalities in DNA methylation are linked to a wide range of health outcomes, including psychiatric disorders [2].

Indeed, over the past two decades, epigenetic research in CAP has collected extensive data that could help resolve the question of how environmental influences can result in the development of CAP disorders. One of the many examples are DNA methylation changes following childhood trauma, which are known to predict emotional and behavioral problems after trauma exposure [15]. Another example are methylation signatures mediating environmental impact in the context of polygenic risk leading to Bipolar Disorder in adolescents [7]. This knowledge of the environmental impact and epigenetic mechanisms has the potential to revolutionize and redefine CAP by enhancing primary and secondary prevention, improving diagnosis, and identifying novel treatment targets. And yet, these insights have up to date little practical value, as they do not effectively contribute to diagnostic processes, identify therapeutic targets, or support the development of personalized medicine by treatment tailoring to individual biological profiles.

So why does this scientific knowledge and data collected not advance CAP clinical and research work as effectively as expected?

We identify three fundamental challenges: (1) the limited connection between our clinical labels and the underlying biological processes; (2) the current approach to conducting epigenetic studies, and, last but not least, (3) the way we generate and analyze Big Data in CAP.

## Clinical labels

can you Imagine enrolling patients in a clinical study of type 2 diabetes treatment based on how subjects feel after a meal!? But this is the way we largely perform CAP research. There is limited relationship between our clinical labels, such as “depression” or “ADHD” and the underlying biological processes. It is highly unlikely that biologically meaningful connections would arise from studies based on ICD or DSM - labels. Without having biologically determined, distinct phenotypes, how can we expect to find consistent relationships between environmental exposures, epigenetic modifications and behavior? This fundamental issue has been to our opinion well addressed by the Research Domain Criteria (RDoC) initiative of the National Institute of Mental Health. RDoC organize the research of mental illnesses, by looking at dimensions of functioning rather than being tied to categorical diagnoses. It is a dynamic structure that currently focuses on six major domains of human functioning: negative and positive valence systems, cognitive systems, social processes, arousal and regulatory systems, and sensorimotor systems. Contained within each domain are several constructs that comprise different aspects of functions and span from “normal to abnormal” with the understanding that each point on this continuum is affected by environmental and neurodevelopmental contexts [11].

We therefore strongly suggest that more CAP studies apply RDoC in order to connect dimensions of functioning with environmental factors and epigenetic data.

## Epigenetic study design

Epigenetic studies in CAP face several methodological challenges, with the most severe being the DNA methylation analysis per se, the establishment of suitable cohorts, and the selection of appropriate sample sources.

DNA methylation can be assessed at different levels, from targeted, gene specific approaches (e.g., pyrosequencing), via “genome scale” approaches (microarray, reduced representation bisulfite sequencing (RRBS)) to full genome wide next-generation sequencing methods. In cohort studies, the current standard is the Illumina 450k or 850k array: It is relatively cheap, has a standardized setup, and there is a wide panel of software available for quality control and data analysis. However, even the large arrays with (roughly) 850k CpG positions only represent a tiny proportion of a typical human epigenome (> 32 million CpG [4]. There is a good chance that we miss important DNA methylation changes because they belong to the roughly 97% of CpG positions not covered by these arrays [12].

Regarding study setup, there are two classic scenarios. Researchers could define a case-control study, which is typically well-characterized with clear clinical definitions. However, the sample size is often small, which leads to the loss of significance when the array data are corrected for multiple testing (i.e., 450 or 850k tests! ). Therefore, the second scenario is often applied, which includes epigenome-wide association studies that are performed involving thousands or hundreds of thousands of participants. This design addresses the issue of multiple testing but introduces bias due to the poorly characterized and heterogeneous sample from multiple study centers, as obtaining comprehensive data on psychiatric conditions, let alone dimensions of functioning, is challenging in large multicenter cohorts.

For obvious reasons studies are usually performed in easy-to-obtain material, for example blood or saliva. However, DNA methylation is in parts cell type specific as it contributes to the specific cellular phenotypes [12]. This implies that data obtained from blood or buccal samples might not represent the situation in our target tissue, somewhere in the brain. Ideally, one needs to test the results in the target tissue as a proof of principle (e.g. Riese et al., [14]. It was proposed to instead focus on “correlated regions of systemic interindividual variation” (CoRSIVs), which do not depend on tissue types [6].

Last but not the least, it is important to consider developmental timing and age effects, as these factors can significantly influence the onset and progression of psychiatric symptoms. For instance, epigenetic changes should be examined before symptom onset, thus providing valuable insights into the early biological markers of mental health issues. Utilizing population-based cohorts with longitudinal assessments of neurobiological changes is essential. By following the same individuals longitudinally from birth, researchers can map the relationship between neurobiological changes, such as epigenetic modifications and psychological symptoms as they unfold over time allowing for a more dynamic understanding of how these neurobiological changes contribute to mental health. Moreover, focusing on broad polyepigenetic signatures can enhance our understanding of complex epigenetic interactions.

## Big data in CAP

CAP research may generate large data sets that encompass environmental, neurobiological, and (epi-) genetic data, collected from a wide variety of sources and formats over various time scales [1, 13]. This massive volume of high-variety data, often referred to as Big Data, serves as a key resource for understanding complex patterns and multifactorial relationships. However, such data can no longer be effectively analyzed using simple methods, such as regression equations [3].

What holds promise in this context are artificial intelligence (AI) systems built upon “data-driven models”, which are machine learning algorithms able to learn and draw inferences from patterns in data (e.g. Wulff & Marschollek [16]. However, AI algorithms are only as good as the data that it is trained on.

So, what does an AI system need to integrate CAP Big Data and make it of use for further research and clinical decisions?

First, these data sets have to be digitally captured and managed precisely. In CAP, not only measurable patient data is incorporated to diagnose but also contextual factors on families, the societal environment, and life events, amongst others. Furthermore, “vague data”, such as speech, feelings or mind sets are very important, but difficult to measure. In the best case, AI systems need to incorporate all of this information, requiring these data sets to be digitally captured and managed precisely. As Big Data technologies advance, from long-term storage to real-time data processing, there are more opportunities to reuse data once it has been collected. Furthermore, the increasing adoption of wearable technologies, such as smartwatches, on the one hand, and patient-reported outcome measures and mobile ecological momentary assessments on the other hand facilitates the collection of rich data sets in everyday life and paves the way for utilizing this information to support remote diagnostic capabilities in CAP.

Second, before using routine data as training data basis for AI algorithms, careful data engineering needs to take place. In particular, terminology and ontology enrichments are required due to the varying use of similar terms in CAP. Data needs to be carefully managed, semantically enriched, and standardized. As standardization in health data management, such as interoperability standards for clinical data and communications like Health Level 7 Fast Healthcare Interoperability Resources, Observational Medical Outcomes Partnership, or open standard electronic health records or advanced terminologies like Systematized Nomenclature of Medicine Clinical Terms, becomes more widely adopted, data will be more harmonizable than it is now.

Third, we will face the challenge of dealing with multi-level, multi-dimensional, and potentially unbalanced data accompanied with heterogeneity, uncertainty, and missingness. With the recent advancements in deep learning, the handling of such data sets has been made possible. With the rise of generative AI algorithms, such as large language models, unstructured data is accessible, processable, and analyzable. The use of foundation models is also promising outside of pure language models, in particular for addressing equifinality and multifocality, since they can process and integrate data from multiple modalities, are high-scalable, and provide extensive contextual learning features. Improvements in variational autoencoders, a class of generative models that learn probabilistic mappings between input data and a structured latent space, offer promising techniques for encoding and disentangling different pathways and combinations of risk factors and outcomes. For unbalanced data, e.g. with respects to gender, age, or occurrence of symptoms and diagnoses it is important to raise an awareness on possible data biases included in the routine data to prevent discriminatory decisions. Technologies can be employed to identify and address these issues in the original data sets before further processing.

## Conclusions

To integrate our recently acquired knowledge of risk and protective environmental factors, along with their impact at the epigenetic level, into our understanding of CAP pathomechanisms and clinical decision-making, fundamental clinical and research issues must be addressed.

First, we strongly recommend that more CAP studies apply RDoC. This will help overcome the obstacles of non-biologically based clinical labels and connect dimensions of functioning with environmental factors and epigenetic data.

Second, it is essential to conduct better-powered, harmonized, multi-cohort studies, which are needed to adequately capture the development of CAP psychopathologies as well as the outcome of environmental exposures and the time-varying nature of DNA methylation. Such studies will provide more robust and generalizable findings.

AI is capable to work with CAP generated Big Data to uncover complex patterns and multifactorial relationships. However, for AI to be applied, the data must be in a form that AI can work with. The data needs to be digitally recorded, managed, and standardized. The importance of routine and so-called ‘vague data’—such as speech, emotions, and mind sets—cannot be underestimated, making a strong case for their digital capture. Therefore, more studies should incorporate wearable technologies, record speech and utilize mobile ecological momentary assessments.

By implementing these steps, we can one day move forward from expert -based to research- and evidence based decision making in CAP.

## References

[1] Boyce WT, Sokolowski MB, Robinson GE (2020) Genes and environments, development and time. Proc Natl Acad Sci U S A 117(38):23235–23241. 10.1073/pnas.2016710117 32967067 10.1073/pnas.2016710117 PMC7519332

[2] Cánepa ET, Berardino BG (2024) Epigenetic mechanisms linking early-life adversities and mental health. Biochem J 481(10):615–642. 10.1042/bcj20230306 38722301 10.1042/BCJ20230306

[3] Dwyer D, Koutsouleris N (2022) Annual research review: translational machine learning for child and adolescent psychiatry. J Child Psychol Psychiatry 63(4):421–443. 10.1111/jcpp.13545 35040130 10.1111/jcpp.13545

[4] Gershman A, Sauria MEG, Guitart X, Vollger MR, Hook PW, Hoyt SJ, Jain M, Shumate A, Razaghi R, Koren S, Altemose N, Caldas GV, Logsdon GA, Rhie A, Eichler EE, Schatz MC, O’Neill RJ, Phillippy AM, Miga KH, Timp W (2022) Epigenetic patterns in a complete human genome. Science 376(6588):eabj5089. 10.1126/science.abj5089 35357915 10.1126/science.abj5089 PMC9170183

[5] Greenberg MVC, Bourc’his D (2019) The diverse roles of DNA methylation in mammalian development and disease. Nat Rev Mol Cell Biol 20(10):590–607. 10.1038/s41580-019-0159-6 31399642 10.1038/s41580-019-0159-6

[6] Gunasekara CJ, Waterland RA (2019) A new era for epigenetic epidemiology. Epigenomics 11(15):1647–1649. 10.2217/epi-2019-0282 31729252 10.2217/epi-2019-0282

[7] Hesam-Shariati S, Overs BJ, Roberts G, Toma C, Watkeys OJ, Green MJ, Pierce KD, Edenberg HJ, Wilcox HC, Stapp EK, McInnis MG, Hulvershorn LA, Nurnberger JI, Schofield PR, Mitchell PB, Fullerton JM (2022) Epigenetic signatures relating to disease-associated genotypic burden in Familial risk of bipolar disorder. Transl Psychiatry 12(1):310. 10.1038/s41398-022-02079-6 35922419 10.1038/s41398-022-02079-6 PMC9349272

[8] Hochberg Z, Feil R, Constancia M, Fraga M, Junien C, Carel JC, Boileau P, Le Bouc Y, Deal CL, Lillycrop K, Scharfmann R, Sheppard A, Skinner M, Szyf M, Waterland RA, Waxman DJ, Whitelaw E, Ong K, Albertsson-Wikland K (2011) Child health, developmental plasticity, and epigenetic programming. Endocr Rev 32(2):159–224. 10.1210/er.2009-0039 20971919 10.1210/er.2009-0039 PMC3365792

[9] Miguel PM, Pereira LO, Silveira PP, Meaney MJ (2019) Early environmental influences on the development of children’s brain structure and function. Dev Med Child Neurol 61(10):1127–1133. 10.1111/dmcn.14182 30740660 10.1111/dmcn.14182

[10] Min JL, Hemani G, Hannon E, Dekkers KF, Castillo-Fernandez J, Luijk R, Carnero-Montoro E, Lawson DJ, Burrows K, Suderman M, Bretherick AD, Richardson TG, Klughammer J, Iotchkova V, Sharp G, Khleifat A, Shatunov A, Iacoangeli A, McArdle A, Relton WL, C. L (2021) Genomic and phenotypic insights from an atlas of genetic effects on DNA methylation. Nat Genet 53(9):1311–1321. 10.1038/s41588-021-00923-x 34493871 10.1038/s41588-021-00923-x PMC7612069

[11] Pacheco J, Garvey MA, Sarampote CS, Cohen ED, Murphy ER, Friedman-Hill SR (2022) Annual research review: the contributions of the RDoC research framework on Understanding the neurodevelopmental origins, progression and treatment of mental illnesses. J Child Psychol Psychiatry 63(4):360–376. 10.1111/jcpp.13543 34979592 10.1111/jcpp.13543 PMC8940667

[12] Pidsley R, Zotenko E, Peters TJ, Lawrence MG, Risbridger GP, Molloy P, Van Djik S, Muhlhausler B, Stirzaker C, Clark SJ (2016) Critical evaluation of the illumina methylationepic BeadChip microarray for whole-genome DNA methylation profiling. Genome Biol 17(1):208. 10.1186/s13059-016-1066-1 27717381 10.1186/s13059-016-1066-1 PMC5055731

[13] Rauh VA, Margolis AE (2016) Research review: environmental exposures, neurodevelopment, and child mental health - new paradigms for the study of brain and behavioral effects. J Child Psychol Psychiatry 57(7):775–793. 10.1111/jcpp.12537 26987761 10.1111/jcpp.12537 PMC4914412

[14] Riese H, van den Heuvel ER, Snieder H, den Dunnen WF, Plosch T, Kema IP, Niezen-Koning KE (2014) Association between methylation of the SLC6A4 promoter region in peripheral blood leukocytes and methylation in amygdala tissue. Psychosom Med 76(3):244–246. 10.1097/psy.0000000000000048 24632895 10.1097/PSY.0000000000000048

[15] van den Oord C, Copeland WE, Zhao M, Xie LY, Aberg KA, van den Oord E (2022) DNA methylation signatures of childhood trauma predict psychiatric disorders and other adverse outcomes 17 years after exposure. Mol Psychiatry 27(8):3367–3373. 10.1038/s41380-022-01597-5 35546634 10.1038/s41380-022-01597-5 PMC9649837

[16] Wulff A, Marschollek M (2018) Learning healthcare systems in pediatrics: Cross-Institutional and Data-Driven Decision-Support for intensive care environments (CADDIE). Stud Health Technol Inf 251:109–112 29968614

